# How do social activities mitigate informal caregivers’ psychological distress? Evidence from a nine-year panel survey in Japan

**DOI:** 10.1186/s12955-016-0521-8

**Published:** 2016-08-22

**Authors:** Takashi Oshio, Mari Kan

**Affiliations:** 1 Institute of Economic Research, Hitotsubashi University, 2-1 Naka, Kunitachi, Tokyo, 186-8603 Japan; 2 School of Economics, University of Hyogo, 8-2-1 Gakuen-Nishi-machi, Nishi-ku, Kobe, Hyogo 651-2197 Japan

**Keywords:** Informal caregiver, Psychological distress, Social activities, Mixed-effects models

## Abstract

**Background:**

It is well known that informal caregiving negatively affects caregivers’ mental health, while social activities improve mental health outcomes among middle-aged and elderly individuals. The goal of the present study was to examine how participation in social activities affected the trajectory of an informal caregiver’s psychological distress.

**Methods:**

We used the data from a nationwide nine-wave panel survey of the middle-aged individuals (aged 50–59 years at baseline) in Japan conducted in 2005–13 (*N* = 24,193 individuals;12,352 women and 11,841 men), mainly focusing on the respondents beginning to provide informal caregiving during the survey period. We employed linear mixed-effects models to explain how the trajectory of psychological distress, measured by Kessler 6 (K6) scores, was associated with caregiving commencement and duration, as well as social activity participation.

**Results:**

Participation in social activities was associated with mitigated K6 scores at caregiving commencement by 66.2 and 58.2 % for women and men, respectively. After caregiving started, participation in social activities reduced the average rise in K6 scores, per year, by 65.6 and 89.6 % for women and men, respectively. We observed similar results when focusing on participation before caregiving commencement to avoid endogeneity problems.

**Conclusion:**

Results suggest that participation in social activities can alleviate caregivers’ psychological distress. Policy measures to support social activities are recommended for the health and well-being of current and potential caregivers.

## Background

It is widely known that informal caregiving is associated with negative mental health outcomes [1–7], especially among women [8–11]. However, evidences regarding the trajectory of mental health outcomes after caregiving commencement are mixed. Several studies suggest that prolonged caregiving causes mental health deterioration [1, 2], while others support an ‘adaptation’ view whereby caregivers are able to adapt to care-related stresses over time even if they are strongly affected by caregiving commencement [12, 13]. Additionally, several factors, such as hours providing care and relationship with the care recipient, confounds caregivers’ mental health trajectories [14, 15].

Meanwhile, several recent studies have provided evidence that social activities, which refer to participation in activities involving interpersonal interactions with others in their neighbourhood, community, or other domains of society–such as volunteering and community engagement–can improve mental health outcomes among middle-aged and elderly individuals [16–22]. Social activities, which are generally beneficial for psychological well-being given that they offer channels for acquiring role support in order to sustain one’s own positive self-concept [18, 19, 22]. Volunteering offers a way of gaining social approval in addition to improving self-esteem [17, 20]. Some studies have focused on social support, which refers to the perception, and/or actuality that one is cared for and has assistance from other people, and its favorable association with mental and physical health outcomes [23–25]. Social activities refer to more active involvement in interactions with others. However, social activities and social support are not mutually exclusive; participating in social activities is expected to increase the chance of receiving actual and/or perceived social support [24].

In the present study, we jointly considered how informal caregiving and social activities could potentially affect mental health outcomes among middle-aged adults using the Japanese data. Specifically, we examined how participation in social activities affected the trajectory of an informal caregiver’s psychological distress. The study took full advantage of a nationwide 9-year panel survey, which allowed the ability to capture when each respondent started caregiving and how long s/he continued providing care, along with changes in psychological distress, participation in social activities, and other conditions.

Given findings from previous studies, we predicted that caregiving would have an adverse impact on caregivers’ mental health, and participation in social activities would mitigate this impact. However, caregiving commencement and duration would likely have different associations with mental health outcomes. Furthermore, the effect of social activity participation may also differ between commencement and the subsequent caregiving years. Moreover, the trajectory of caregivers’ mental health in relation to social activity participation may differ by gender. We explicitly addressed these issues in the present study.

## Methods

### Study design

We conducted longitudinal analyses that aimed to examine how (i) caregiving commencement and (ii) its duration were associated with caregiver psychological distress and participation in social activities, using panel data obtained from a nationwide, population-based survey. We constructed two sample subsets to examine the associations of (i) and (ii) separately with caregiver psychological distress, and employed linear mixed-effects models to examine the trajectory of caregiver psychological distress.

### Study sample and procedure

Nine waves of panel data were obtained from a nationwide, population-based survey (The Longitudinal Survey of Middle-Aged and Older Adults), which was conducted by the Japanese Ministry of Health, Labour and Welfare (MHLW) each year between 2005 and 2013. Samples from the first wave were limited to individuals aged 50–59 years and were collected in November 2005 via a two-stage random sampling procedure. First, 2515 districts were randomly selected from 5280 districts used in the MHLW’s nationwide, population-based “Comprehensive Survey of the Living Conditions of People on Health and Welfare (CSLCPHW)” conducted in 2004. The 5280 districts, originally selected for the CSLCPHW, were randomly selected from about 940,000 national census districts. Second, 40,877 residents aged 50–59 years as of October 30, 2005 were randomly selected from each selected district, according to its population size. The questionnaires were physically distributed to the participants’ homes, where they were completed by the participants as of November 2, and physically collected several days thereafter.

A total of 34,240 individuals responded (response rate: 83.8 %). The second to ninth waves were conducted in early November of each year from 2006 to 2013. The number of respondents declined gradually to 23,722 at the ninth wave (attrition rate from the first wave: 19.0 %). No new respondents were added after the first wave. We excluded respondents who were missing data on key variables, but we did not apply any specific inclusion/exclusion criteria for this study.

### Sample datasets

We constructed two sample subsets. The first subset consisted of respondents who had not cared for a family member during the year prior, in order to examine how caregiving commencement was associated with psychological distress. We excluded those who had already provided care during the year prior in order to identify whether the respondent actually “started” caregiving at each wave. We excluded all data from wave 1, because we did not know the respondents’ prior caregiving status.

The second subset consisted of respondents who started caregiving between waves two and eight, and provided care during the second year or later, to examine how caregiving duration was associated with psychological distress. As with the first subset, we excluded those who provided care during wave 1, because we could not know how long they had been providing care up to that point. We also excluded the data for caregiving commencement, because we could not distinguish between the impact of caregiving commencement and its duration.

**Fig. 1:**
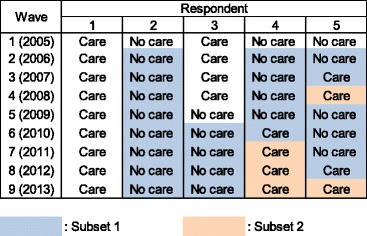
Constructing two sample subsets (subsets 1 and 2): illustrative examples

The process of the construction of the two datasets is illustrated in Fig. 1, which provides examples showing each respondent’s caregiving status (“Care” or “No care”) at each wave and indicates how to allocate the data for each respondent-wave combination to subsets 1 and 2. For all respondents, the data from wave 1 were excluded in both subsets, because we could not determine their prior caregiving status. Respondent 1 cared throughout nine waves, but we did not use her data at all because we could not precisely identify her caregiving duration; she may have started caregiving before wave 1. By contrast, respondent 2 provided no care at all during the sample period. We allocated her data from waves 2 to 9 to subset 1, because we could determine whether she started caregiving or not at each wave. For respondent 3, who kept caregiving until wave 4, we excluded her data until wave 4, because there was no precise information about her caregiving duration. We excluded her wave 5 data, when she stopped caregiving, because the association between caregiving termination and caregiver mental health was not addressed in this study. Respondent 4 started caregiving at wave 6 and continued this until wave 9. We allocated her data to subset 1 for waves 2 to 6, and to subset 2 for waves 7 to 8. Finally, respondent 5 showed a non-continuous pattern of caregiving. We allocated her data into subsets 1 and 2 based on the abovementioned rules, without any special treatment.

In the current dataset, these two subsets comprised 140,401 observations (for 28,173 individuals) and 7337 observations (for 3164 individuals), respectively. A total of 6279 individuals (3591 women and 2688 men) commenced caregiving during waves two and eight.

### Measures

#### Informal caregiving

We used two variables to assess caregiving status. The first was a binary variable to which we allocated a ‘1’ if the respondent started providing care to at least one family member, regardless of their living arrangements. The second was a continuous variable of years (from 1 to 7) after the start of caregiving. We further considered four care-related factors. First, we assessed the number of family members to whom a respondent provided care and constructed two binary variables of (1) two and (2) three or more, respectively. Second, we constructed a binary variable to indicate residing with a care recipient (at least one recipient if a respondent provided care to more than two). Third, we considered hours of care. The survey asked respondents how many hours they spent on caregiving tasks per week. We constructed three binary variables corresponding to second, third, and fourth quartiles of reported hours of care (with 3, 8, and 21 h per week as thresholds).

#### Social activities

Respondents were asked whether they participated in six types of social activities (hobbies or cultural activities, exercise or sports, community events, support for children, support for the elderly, and ‘other’ activities) within the past year from the date of the survey. These questions, which were asked separately from those about their family activities such as informal care for family members and other housework, aimed to determine respondents’ participation in activities involving interpersonal interactions with others mainly in their neighbourhood or community. Those who participated in any of social activities were also asked to indicate the manner in which they participated (alone, with family or friends, with co-workers [including former co-workers], in a neighbourhood community association, or in a non-profit organization or corporation in the public interest). An individual was considered to have participated in a social activity if s/he participated in at least one of the four types; we constructed a binary variable corresponding to this definition.

#### Psychological distress

We measured psychological distress using K6 scores [26, 27]. Respondents were asked to complete a six-item psychological distress questionnaire: ‘During the past 30 days, about how often did you feel a) nervous, b) hopeless, c) restless or fidgety, d) so depressed that nothing could cheer you up, e) that everything was an effort, or f) worthless?’ Responses were rated on a 5-point scale (0 = none of the time to 4 = all of the time). A summed score (range: 0–24) was calculated. Higher K6 scores reflect higher levels of psychological distress. The distribution of K6 scores is generally skewed toward lower values. Thus, in the present study, we focused on changes in (rather than levels of) K6 scores from prior to and after caregiving commencement.

#### Covariates

We controlled for several sociodemographic factors: age at the first wave, educational attainment (graduated from junior high school, high school, junior college, college or above, and other), marital status (having a spouse or not), and employment status. We also controlled for household expenditures as a proxy for household income, considering that the survey only asked respondents about their own and their spouse’s income, and more respondents reported household expenditures rather than income. We adjusted household expenditures based on household size by dividing expenditure amount by the square root of the number of household members. We further controlled for wave-specific factors by including binary variables at each wave.

### Statistical analyses

For longitudinal analyses, we employed linear mixed-effects models, which can capture both between-individual and within-individual variations [1, 2, 15, 28]. Mixed-effects models are analogous to multilevel models, which assume that observations at each wave are nested within an individual.

We estimated four types of linear mixed-effects models to explain changes in psychological distress. Models 1–3 considered how psychological distress was impacted by caregiving commencement. As a benchmark model, Model 1 assessed changes in K6 scores by caregiving commencement, controlling for pre-caregiving K6 scores and the covariates. Model 2 added the interaction term between caregiving commencement and participation in social activities to Model 1.

It is reasonable to hypothesize that caregiving commencement would exacerbate psychological distress—that is, the estimated coefficient of caregiving commencement would be positive—given that previous studies have generally shown a negative association between caregiving and mental health [1–11]. We also predicted that social support would mitigate the adverse impact of caregiving commencement on psychological distress, in accordance with results from previous studies [16–22]. If this prediction is correct, the sign of the interaction term for caregiving commencement and participation in social activities would be positive.

A possible concern could be the endogeneity of social activity participation. For instance, it might be possible that caregiving commencement could discourage caregivers from being (or even encouraged to be) socially active, leading to biased estimation results. To address this issue and examine the robustness of Model 2, Model 3 we used participation in social activities 1 year prior to caregiving commencement as a regressor.

Based on results from Models 2 and 3, we computed the proportion of the estimated coefficient for the interaction term between caregiving commencement and social activity participation to that for caregiving commencement. This proportion assessed the extent to which participation in social activities mitigated the negative impact of caregiving commencement on caregivers’ mental health.

Models 4–6 examined changes in K6 scores during subsequent years after caregiving commencement, focusing on those who had started caregiving during waves two and eight. These models aimed to explain changes in K6 scores based on the time since caregiving commencement and its interaction with caregivers’ participation in social activities. Models 4–6 were counterparts to Models 1–3, respectively. Model 4 used the number of caregiving years as a key explanatory variable. The estimated coefficient can be interpreted as an average rise in K6 scores per year. Model 5 added the interaction term between the number of caregiving years and participation in social activities. Model 6 included participation in social activities 1 year prior to caregiving commencement. These models controlled for K6 scores at caregiving commencement, as well as the other variables used in Models 1–3. The estimated coefficients for the number of caregiving years are comparable with those for caregiving commencement obtained in Models 1–3. Unlike caregiving commencement, it is difficult to predict how caregiving duration would be associated with psychological distress, given mixed results from previous studies [1, 2, 12, 13]. However, we expected to observe a favorable confounding effect of participation in social activities. In Models 5 and 6, we computed the proportion of the estimated coefficient for the interaction term between caregiving years and participation in social activities to that for caregiving years. This proportion indicated the extent to which caregiving commencement mitigated the negative association between caregiving duration and caregivers’ mental health.

## Results

### Descriptive analyses

Table 1 summarizes key sociodemographic variables at baseline (in 2005). 9.1 % of the women and 5.4 % of the men provided informal care, and 72.8 % of the women and 70.6 % of the men participated in social activities. Hence, women were both more involved in informal care and socially active. The bottom part of the table summarizes the maximum caregiving duration. 71.3 % of the women and 77.8 % of the men had no experience with caregiving during the survey period. About half of the caregiver sample was a caregiver at commencement, and the proportion of caregivers in the sample declined as the survey proceeded. Six-point-one percent of the women and 4.9 % of the men had non-continuous caregiving experiences, after starting caregiving at wave 2 or later.

**Table 1:** Key sample characteristics at baseline (in 2005)

	All		Women		Men	
	*M*	(*SD*)	*M*	(*SD*)	*M*	(*SD*)
Age (years)	54.7	(2.7)	54.6	(2.7)	54.7	(2.7)
Educational attainment (%)						
Junior high school	16.2		15.6		16.8	
High school	58.4		64.1		52.5	
Junior college	7.6		12.4		2.7	
College	17.1		7.4		27.3	
Other	0.6		0.5		0.7	
Having a spouse (%)	87.6		86.2		88.9	
Number of household members	2.5	(1.3)	2.4	(1.3)	26	(1.3)
Employed (%)	81.7		69.3		94.5	
Household spending^a^	230.8	(220.6)	224.8	(191.0)	237.1	(247.6)
Informal caregiving (%)	7.3		9.1		5.4	
Participated in social activities (%)	71.7		72.8		70.6	
K6 score (range: 0–24)	3.0	(3.9)	3.2	(4.0)	2.8	(3.9)
Maximum caregiving duration (% of total [% of caregivers])^b^						
No care	74.5		71.3		77.8	
1 year (commencement)	12.8	[50.1]	13.5	[47.0]	12.2	[54.3]
2 years	5.2	[20.2]	5.7	[19.7]	4.6	[20.9]
3 years	3.0	[11.8]	3.7	[12.8]	2.3	[10.4]
4 years	1.9	[7.3]	2.2	[7.7]	1.5	[6.6]
5 years	1.1	[4.5]	1.6	[5.6]	0.7	[3.0]
6-8 years	1.6	[6.2]	2.0	[7.1]	1.1	[4.9]
(Non-continuous caregiving	5.4	[21.2]	6.1	[21.3]	4.9	[20.9])
Number of individuals	24,193		12,352		11,841	

^a^Monthly, thousand yen, adjusted for household size

^b^Due to rounding, percentages do not always add up to 100 %

**Fig. 2:**
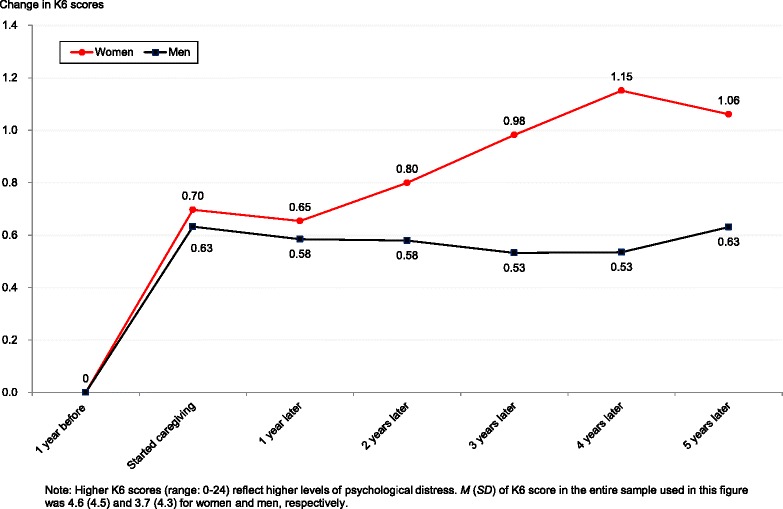
Comparing changes in K6 scores from the pre-caregiving level between female and male caregivers

Figure 2 depicts changes in K6 scores for both the men and women, plotting average changes from the year prior to caregiving commencement. Caregiving commencement caused an increase in K6 scores for both women and men, confirming an initial rise in distress. However, the figure indicates that the trajectory of K6 scores across subsequent years differed substantially between women and men. For women, K6 scores showed a general, monotonic increase—albeit with a much more moderate slope after the initial rise. This confirms the notion that prolonged caregiving could lead to increased psychological distress. In contrast, K6 scores remained relatively stable for men, suggesting that mental health outcomes were not sensitive to prolonged caregiving. The differences in the trajectory of K6 scores between women and men will be discussed more rigorously later, based on the regression results.

**Fig. 3:**
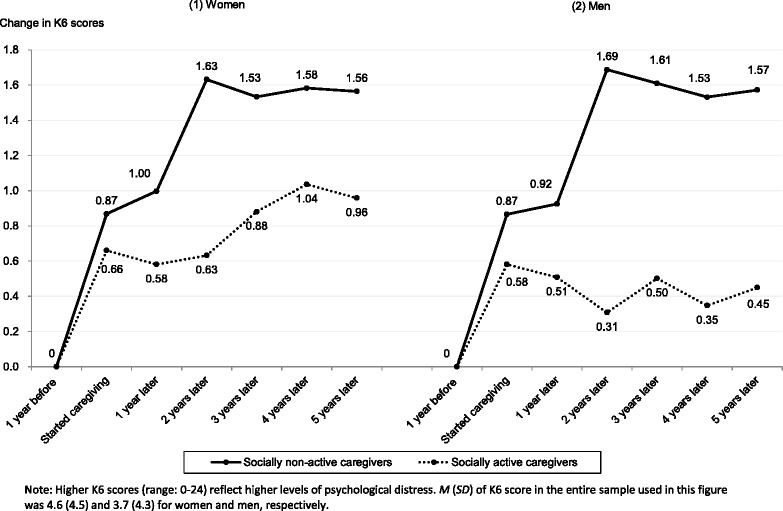
Comparing changes in K6 scores from the pre-caregiving level between socially active and non-active caregivers

Figure 3 compares the trajectory of K6 score changes between socially active and non-active caregivers for women and men. We observed similar patterns for both genders. The rise in K6 scores at caregiving commencement was somewhat larger for those who were not participating in social activities. Furthermore, the K6 score slopes were a bit steeper for non-participants. For men, the K6 score slope is even negative, albeit slightly, for participants; the slope is positive for non-participants. A stable slope observed for men in Fig. 2 obscures this discrepancy.

To help assess the validity of these arguments, we estimated regression models to explain the trajectory of K6 scores. Table 2 examined determinants of K6 score changes at caregiving commencement, focusing on respondents who did not provide care 1 year prior, for women and men. Model 1, which used the binary variable of caregiving commencement as a single key regressor, shows that K6 scores increased at caregiving commencement for both women and men. The negative coefficient on pre-caregiving K6 scores indicates that an increase in K6 scores was more limited for those with already higher scores.

**Table 2:** Estimated associations in K6 score changes based on caregiving commencement (using sample subset 1)^a^

Dependent variable: change in K6 score from pre-caregiving commencement	Model 1		Model 2		Model 3	
	*β*	(SE)	*β*	(SE)	*β*	(SE)
Women (*N*. of observations [individuals] = 69,885 [14,356])						
Caregiving commencement (A)	0.32***	(0.09)	0.75***	(0.14)	0.56***	(0.13)
× Participated in social activities (B)			−0.50***	(0.12)		
[− (B)/(A) %]			[66.2***	(*10.4*)]		
× Participated in social activities prior to caregiving commencement (C)					−0.30**	(0.12)
[− (C)/(A) %]					[53.7***	(*14.6*]
K6 score one year before caregiving commencement	−0.64***	(0.01)	−0.64***	(0.01)	−0.64***	(0.01)
Men (*N*. of observations [individuals] = 70,516 [13,817])						
Caregiving commencement (A)	0.41***	(0.10)	0.80***	(0.15)	0.69**	(0.14)
× Participated in social activities (B)			−0.47***	(0.13)		
[− (B)/(A) %]			[58.2***	(*11.1*)]		
× Participated in social activities prior to caregiving commencement (C) –0.35					−0.35**	(0.13)
[− (C)/(A) %]					[50.3***	(*13.0*)]
K6 score one year before caregiving commencement	−0.70***	(0.01)	−0.70***	(0.01)	−0.70***	(0.01)

^a^Controlled for age at the first wave (2005), educational attainment, marital status, household spending (adjusted for household size), employed, number of care recipients, hours of care, residing with a care recipient, and waves. The full results are available upon request

***p < 0.001, **p < 0.01, *p < 0.05

In Model 2, which added the interaction term between caregiving commencement and participation in social activities, we observed that the interaction term was negatively associated with K6 scores. By dividing the interaction coefficient by the caregiving commencement coefficient, we observed that participation in social activities reduced the adverse impact of caregiving commencement on K6 scores by 66.2 % for women and 53.7 % for men. We confirmed a similar, albeit somewhat smaller, mitigating effect of participation in social activities when using pre-caregiving participation in social activities for Model 3.

Table 3 presents how caregivers’ K6 scores changed over time after caregiving commencement. We used the number of caregiving years as a key regressor. Model 4, which showed general associations between caregiving duration and K6 scores, underscores the findings shown in Fig. 2. First, the duration coefficient was much smaller than the commencement coefficient observed in Table 2, which is consistent with the fact that K6 score slopes became much more moderate after caregiving commencement. Second, the duration coefficient was significant and positive for women but non-significant for men. Again, this is consistent with the fact that the K6 curve was positively sloped for women but almost flat for men.

**Table 3:** Estimated associations of K6 score changes based on caregiving duration (using sample subset 2)^a^

Dependent variable: change in K6 score from pre-caregiving commencement	Model 4		Model 5		Model 6	
	*β*	(SE)	*β*	(SE)	*β*	(SE)
Women (*N*. of observations (individuals) = 4637 (1.922))						
Duration years from caregiving commencement (A)	0.11**	(0.04)	0.23***	(0.05)	0.19***	(0.05)
× Participated in social activities (B)			−0.15***	(0.04)		
[− (B)/(A) %]			[65.6***	(*13.7]*]		
× Participated in social activities prior to caregiving commencement (C)					−0.11*	(0.04)
[− (C)/(A) %]					[*56.8* ^**^	(*18.2*)]
K6 score at caregiving commencement	−0.32***	(0.01)	−0.33***	(0.01)	−0.33***	(0.01)
Men (*N*. of observations (individuals) = 2700 (1242))						
Duration years from caregiving commencement (A)	0.05	(0.05)	0.20**	(0.06)	0.19**	(0.07)
× Participated in social activities (B)			−0.18***	(0.05)		
[− (B)/(A) %]			[89.6***	(*23.9*)]		
× Participated in social activities prior to caregiving commencement (C)					−0.18**	(0.06)
[− (C)/(A) %]					[95.3***	(*27.4*)]
K6 score at caregiving commencement	−0.34***	(0.02)	−0.34***	(0.02)	−0.34***	(0.02)

^a^Controlled for age at the first wave (2005), educational attainment, marital status, household spending (adjusted for household size), employed, number of care recipients, hours of care, residing with a care recipient, and waves. The full results are available upon request

***p < 0.001, **p < 0.01, *p < 0.05

Model 5, which added the interaction term between duration and participation in social activities, revealed a significant and negative association for both genders. Participation in social activities reduced the impact of prolonged caregiving by 65.6 % for women and 89.6 % for men. For men, a significant positive association between prolonged caregiving and K6 scores, which was not observed in Model 4, was largely offset by participation in social activities. Model 6, which included pre-caregiving participation in social activities, provided similar results. We found no significant impact of resumed caregiving after a break on caregiver psychological distress, after adding an indicator variable for resumed caregiving and/or its interaction term with caregiving to the predictors (not reported in Table 3).

## Discussion

### Main findings

The present study examined how informal caregivers’ mental health is associated with caregiving and participation in social activities. Based on a nationwide panel survey, we examined the impacts of caregiving commencement and duration separately. We obtained three key results. First, we confirmed that informal caregiving had an adverse impact on caregivers’ mental health in terms of psychological distress, similar with several previous studies [1–7]. Studies from Europe have revealed a clear North–south gradient [29, 30]; compared to northern countries, the provision of informal care has a more negative impact on caregivers’ mental health in southern (Mediterranean) countries, where informal care is still the main source of long-term care (LTC) support services. Our findings suggest that Japan aligns with these ‘southern’ countries, especially Japanese women.

Second, we observed that the impact of caregiving commencement and duration on mental health differs substantially. For instance, commencement led to a stark increase in psychological distress for both women and men. However, duration was associated with a moderate rise in women’s distress, while men’s distress was largely insensitive to prolonged caregiving. Many preceding studies have found that caregiving tends to have a more adverse association with mental health for women than men [8–11]. Results from the present study showed that this gender difference was remarkably strong with prolonged caregiving.

Third and most importantly, we found that participation in social activities substantially mitigated the negative impact of caregiving on mental health. This is in line with results from previous studies revealing that participation in social activities has a favourable effect on mental health outcomes [16–22]. We also observed that participation in social activities was more important for men. As noted above, we observed that male caregivers were generally insensitive to prolonged caregiving. However, we revealed a remarkable difference between social participants and non- participants among male caregivers. Participating in social activities largely offset the negative impact of prolonged caregiving for men, and this was not necessarily the case for non-participants. The prolonged effect of caregiving distress may only be mitigated for men who are socially active and engaged.

### Practical implications

Results from the present study have important practical implications. First, we confirmed that informal caregiving is potentially a major risk factor for middle-aged individuals in Japan. The Japanese LTC system depends heavily on informal care at home, although a public LTC insurance system was initiated in 2000 to relieve family caregivers of the burdens associated with their roles [31]. Indeed, recent statistics show that more than 70 % of LTC is provided at home [32], and its burden falls heavily upon women within Japan’s multigenerational family structure [33].

Second, based on the results from the present study we can reasonably argue that social activities may be a protective factor against poor mental health among both current and potential caregivers. Thus, policy support should be provided to encourage social activities for middle-aged caregivers. Informal caregivers should not be isolated from interpersonal interactions in their neighbourhood, community, or other domains of society to avoid the adverse impact of prolonged caregiving on mental health.

### Strengths and limitations of the study

There are at least two possible criticisms for this interpretation, both of which are related to endogeneity issues. First, it could be possible that participating in social activities is affected by caregiving, which likely reduces chances to participate in social activities or even encourages caregivers to be socially active to look for assistance and support. Thus, to avoid potential biases due to reverse causation, we predicted K6 scores by pre-caregiving participation in social activities and obtained similar results.

Second, we did not remove the possibility that certain unobserved individual attributes, such as personality traits, intelligence, and other inherent characteristics, may produce a greater inclination to participate in social activities (especially if those activities are voluntary). Thus, certain individual attributes – rather than participation in social activities itself – may encourage individuals to participate in social activities, and this might be what mitigates the adverse impact of caregiving on mental health. We recognize that the present analytic framework could not distinguish between these two possibilities. Despite this limitation, the present results underscore that non-participation in social activities is a reliable signal for potential, negative mental health outcomes resulting from caregiving distress.

Besides these two criticisms, several issues remain to be addressed. First, we did not take into account the kin relationships between the caregiver and care recipient, which are likely to affect the trajectories of caregiver psychological distress [33, 34]. Second, we did not consider care levels of care recipients, utilization of formal support from the LTCI program, or informal support from the caregiver’s family members or friends, due to limited data availability, although all of these are potential confounders for the association between caregiving and caregiver mental health. Third, we did not incorporate the simultaneity of caregiver decisions about caregiving, work, residence, and other living arrangements into the regression models. These issues should be addressed in future research to acquire an in-depth knowledge about the dynamics of caregiver mental health.

## Conclusions

Participation in social activities substantially mitigated the adverse impact of caregivers’ mental health, both at caregiving commencement and during subsequent years. We obtained similar results when focusing on pre-caregiving activity participation so as to avoid endogeneity issues. We can conclude that social activities are expected to play an important role for caregiver mental health especially if the LTC system remains heavily dependent on informal care at home. Results also suggest panel studies are needed to analyze the dynamic determinants of caregiver mental health.

## Abbreviations

CSLCPHW: Comprehensive Survey of the Living Conditions of People on Health and Welfare
K6: Kessler psychological distress scale
MHLW: Ministry of Health, Labour and Welfare
